# Programmed cell death in hepatocellular carcinoma: mechanisms and therapeutic prospects

**DOI:** 10.1038/s41420-024-02116-x

**Published:** 2024-08-08

**Authors:** Xiang’an Wu, Jingying Cao, Xueshuai Wan, Shunda Du

**Affiliations:** 1 Department of Liver Surgery, Peking Union Medical College Hospital, PUMC and Chinese Academy of Medical Sciences, Dongcheng, Beijing 100730 China; 2 Zunyi Medical University, Zun Yi, Guizhou 563000 China

**Keywords:** Cell death, Oncogenesis, Cancer therapeutic resistance

## Abstract

Hepatocellular Carcinoma (HCC), the most common primary liver cancer, ranks as the third most common cause of cancer-related deaths globally. A deeper understanding of the cell death mechanisms in HCC is essential for developing more effective treatment strategies. This review explores programmed cell death (PCD) pathways involved in HCC, including apoptosis, necroptosis, pyroptosis, ferroptosis, and immunogenic cell death (ICD). These mechanisms trigger specific cell death cascades that influence the development and progression of HCC. Although multiple PCD pathways are involved in HCC, shared cellular factors suggest a possible interplay between the different forms of cell death. However, the exact roles of different cell death pathways in HCC and which cell death pathway plays a major role remain unclear. This review also highlights how disruptions in cell death pathways are related to drug resistance in cancer therapy, promoting a combined approach of cell death induction and anti-tumor treatment to enhance therapeutic efficacy. Further research is required to unravel the complex interplay between cell death modalities in HCC, which may lead to innovative therapeutic breakthroughs.

## Facts

- Programmed cell death plays an important role in the development and progression of hepatocellular carcinoma. Current anti-tumor strategies exert tumoricidal effects through various programmed cell death pathways.
- The most extensively studied programmed cell death modalities in hepatocellular carcinoma encompass apoptosis, necroptosis, pyroptosis, ferroptosis, and immunogenic cell death, which are interrelated and exert reciprocal effects upon one another.
- Targeting critical molecules in programmed cell death pathways, or synergizing them with anti-tumor drugs, can potentiate anticancer efficacy and provide a promising route for hepatocellular carcinoma therapy.

## Questions

- How do the various types of programmed cell death occur in hepatocellular carcinoma?
- How are different types of programmed cell death interconnected in hepatocellular carcinoma?
- Which form of programmed cell death plays a primary role in the development of hepatocellular carcinoma?
- How might the integration of programmed cell death induction with other therapeutic modalities amplify the anti-tumor activity in hepatocellular carcinoma?

## Introduction

Hepatocellular carcinoma (HCC) is the predominant form of primary liver cancer and the third most frequent cause of cancer-related mortality worldwide, with a 5-year survival rate of only 18% [1, 2]. Currently, surgery is the only curative treatment for HCC, while alternative therapies, such as targeted therapy and immunotherapy, are limited by low response rates and a tendency for drug resistance [3, 4]. Enhancing the effectiveness of HCC treatment requires a thorough understanding of its pathophysiology. The development of HCC is complex and influenced by multiple factors such as environmental, genetic, and immune factors [5]. Various cell death responses, including both programmed and non-programmed pathways, play a critical regulatory role in HCC progression [6, 7]. Fostering selective cell death is a viable approach for the targeted elimination of liver cancer cells.

Cell death is predominantly classified into two types: accidental and programmed cell death (PCD) [8]. Accidental cell death occurs without discernible regulatory mechanisms or signals, whereas PCD operates under stringent molecular and signaling control [9]. Since the discovery of apoptosis in 1972, many new forms of non-apoptotic PCD have been identified and extensively studied, including lysosome-dependent cell death (LCD), pyroptosis, NETosis, immunogenic cell death (ICD), necroptosis, entosis, parthanatosis, ferroptosis, autosis, alkaliptosis, oxiptosis, cuproptosis, and disulfidptosis [10, 11]. Although these PCD processes vary, most are mediated by caspases and oxidative stress, which are often regulated by receptor-interacting protein kinase-1 (RIPK1) [9]. Research reveals that most PCD forms are integral to the pathophysiology of HCC, significantly influencing its development and progression. Apoptosis, necroptosis, pyroptosis, ferroptosis, and ICD have been extensively examined in patients with HCC. However, the predominant cell death signals that drive HCC progression remain a topic of scientific inquiry, underscoring the importance of continuous research into the regulation of PCD to manage disease progression. This review explores the pathological mechanisms and potential regulatory factors within PCD pathways in HCC and explores current and emerging therapeutic interventions that leverage these cell death pathways.

### Hepatocellular carcinoma and apoptosis

#### Molecular pathways of apoptosis

Apoptosis, the earliest described form of PCD, is characterized by cytoplasmic shrinkage, nuclear condensation, fragmentation, and the emergence of apoptosomes. This process is divided into intrinsic and extrinsic pathways, with the former centered on mitochondrial signals and the latter on death receptor engagement [12]. Within the intrinsic pathway, the BCL-2 protein plays a crucial role by binding to Bcl-2 homology-3 (BH3)-only proteins, precipitating the activation of BCL-2-associated X protein (BAX) or BCL-2 antagonist/killer (BAK), thereby inducing mitochondrial outer membrane permeabilization and the consequent release of pro-apoptotic factors, ultimately leading to caspase activation, protein disaggregation, and cellular demise [13]. In contrast, the extrinsic pathway is initiated by membrane-bound death receptors, notably tumor necrosis factor receptor superfamily member 6 (FAS) and tumor necrosis factor receptor-1 (TNFR1), which trigger caspases 8 and 10 [14].

**Fig. 1:**
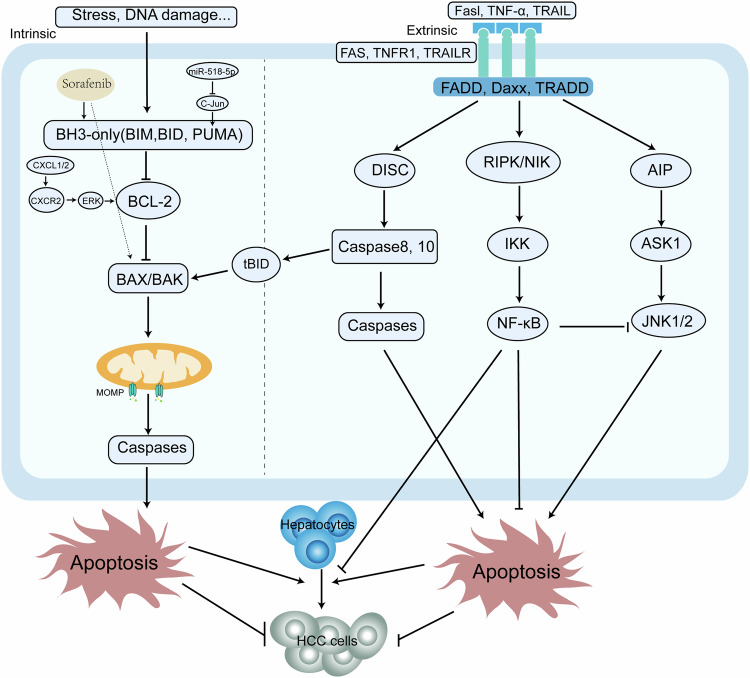
Intrinsic and extrinsic apoptosis in HCC. Intrinsic apoptosis is triggered by stress or DNA damage, activating BH3-only proteins that bind with BCL-2 proteins to form BAX/BAX oligomers, leading to mitochondrial permeability and the release of apoptotic factors. In HCC, it is regulated by signaling pathways like CXCL1/2-CXCR2-ERK. The extrinsic pathway is mediated by death receptor signaling and specific death ligands, forming trimers that activate caspase cascades, IKK/NF-κB, and JNK pathways. The IKK/NF-κB pathway promotes cell survival and inhibits JNK-mediated apoptosis. tBID from the extrinsic pathway can bind with BAX and BAK1 to form MOMP. Apoptosis promotes HCC formation but inhibits it afterward. Drugs like sorafenib can promote apoptosis for anti-tumor effects.

Furthermore, the Nuclear factor-kappa B (NF-κB) pathway has been implicated in the inhibition of apoptosis and oncogenesis of HCC. Tumor Necrosis Factor-alpha (TNF-α) may govern apoptosis by binding to proteins such as inhibitors of kappa B kinase (IKK), c-Jun N-terminal kinase (JNK), and caspases. Moreover, NF-κB activation can repress caspase activity by modulating IKK and counteract TNF-α-induced JNK activation, resulting in apoptotic inhibition [15]. IKK2-mediated NF-κB activation can prevent liver cell damage and hinder HCC development, yet the loss of factors such as RIPK1 and tumor necrosis factor receptor-associated factor 2 (TRAF2) may lead to inhibited NF-κB activity and subsequent spontaneous development of HCC [16, 17]. However, anomalous NF-κB activation in existing HCC can propel HCC progression and promote chemotherapeutic resistance [18, 19] (Fig. 1).

#### Apoptosis dysregulation and drug resistance

In HCC, some anti-tumor drugs induce apoptosis in cancer cells, and the absence of certain molecules within the apoptotic pathway can lead to drug resistance. Addressing these molecules holds promise for restoring the sensitivity of HCC cells to treatment. For instance, sorafenib has been shown to stimulate BAX/BAK in liver cancer cells, largely via the BCL-2 homology-3 domain-only (BH3-only) protein p53-upregulated modulator of apoptosis (PUMA), thus promoting the apoptosis of tumor cells [20]. Moreover, C-X-C motif chemokine ligand-1 (CXCL1) and CXCL2 are known to affect the expression of BCL-2 family genes through the C-X-C chemokine receptor-2 (CXCR2)/extracellular regulated protein kinase (ERK) signaling pathway, which enhances HCC cell activity and reduces the apoptotic effect of sorafenib, thereby contributing to drug resistance. Accordingly, targeting CXCL1, CXCL2, and the subordinate CXCR2/ERK signaling pathway may represent an efficacious strategy to overcome this resistance in HCC [21]. Additionally, MiR-518d-5p has been implicated in fostering sorafenib resistance by interfering with C-Jun/PUMA-mediated apoptosis, with elevated serum levels of MiR-518d-5p correlating with diminished duration and survival rates after sorafenib treatment [22].

Hence, dysregulation of apoptosis may play a role in the development of HCC. The NF-κB pathway, vital in suppressing apoptosis and tumorigenesis, can paradoxically encourage tumor cell survival and drug resistance following the onset of HCC. Modulating components of both the apoptosis pathway and the NF-κB pathway may, therefore, augment the treatment responsiveness of HCC (Fig. 1).

### Hepatocellular carcinoma and necroptosis

#### The role of necroptosis in tumor progression

Necroptosis is induced through the activation of RIPK3 by factors such as death receptors, Toll-like receptors (TLRs), or viruses via RIPK1, Toll-like receptor adapter molecule 1 (TICAM1), and Z-DNA binding protein 1 (ZBP1). This, in turn, activates the mixed lineage kinase domain-like protein (MLKL), leading to the rupture of the plasma membrane and the release of intracellular contents [23].

Necroptosis is associated with chronic liver inflammation, which can progress to fibrosis, chronic liver disease, and potentially HCC [24, 25]. Liver cells with attenuated RIPK3 expression may undergo a sublethal necroptotic process upon parallel NF-κB activation, resulting in cytokine release that can facilitate HCC progression. Conversely, inhibiting NF-κB may tip the balance towards lethal necroptosis, precluding cytokine release and HCC onset [26]. In HCC, RIPK3 downregulation within macrophages correlates with increased M2 tumor-associated macrophage (TAM) accumulation and polarization, contributing to HCC tumorigenesis [27]. Conversely, increased sorbitol dehydrogenase (SORD) levels in HCC appear to bolster necroptotic signaling, encourage M1 macrophage polarization within the tumor milieu, and possibly restrain tumor growth [28]. Moreover, necroptosis in HCC is related to tumor-infiltrating lymphocytes, especially CD8 + T cells, in tumors expressing necroptosis-related genes [29]. Additionally, studies have indicated that recombinant adenovirus (rAAV) induces acute liver damage, necroptosis, and HCC in diabetic and obese mice via the phosphatidylethanolamine-binding protein 1 (Pebp1) pathway, indicating that it is a potential HCC risk factor in these populations [30].

**Fig. 2:**
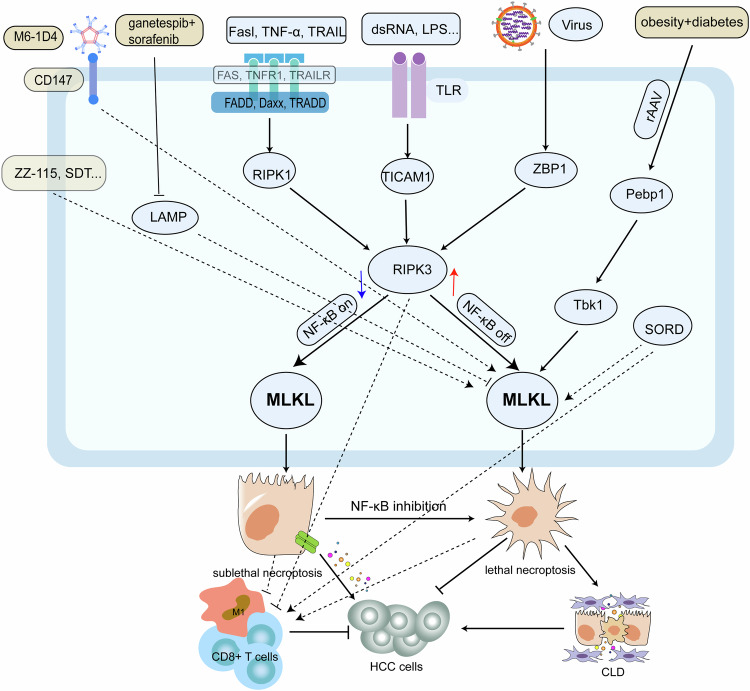
The necroptosis mechanism in HCC. Necroptosis is triggered by death receptors (FAS, TNFR1, TRAILR), TLR, or viruses, which activate RIPK3 through RIPK1, TICAM1, and ZBP1, leading to MLKL activation. This causes the plasma membrane to break and release cell contents. Necroptosis can hinder HCC cell survival but may cause chronic inflammation in hepatocytes. Lower levels of RIPK3 can activate a nonlethal necroptotic NF-κB pathway that can promote HCC progression. Necroptosis also influences HCC development through the regulation of TME and can be influenced by drugs or other signaling molecules.

Necroptosis thus plays a paradoxical role in liver pathology; it fosters inflammation, fibrosis, and HCC evolution; however, in existing HCC, it can also invoke tumor cell death and exert a tumoricidal effect by modulating the necrotic tumor microenvironment (TME), including macrophage polarization and CD8 + T cell infiltration (Fig. 2).

#### Necroptosis stimulation and enhanced anti-tumor drug efficacy

The necroptosis pathway and its associated mechanisms offer substantial potential for HCC therapy. Sorafenib reduces microvessel density, leading to intratumoral hypoxia, which in turn activates hypoxia-inducible factor (HIFs)-mediated cellular reactions, including an elevation of heat shock protein 90 (HSP90). HSP90 interacts with the RIPK1/RIPK3/MLKL complex to facilitate chaperone-mediated autophagic degradation, believed to be the primary cause of sorafenib resistance. Ganetespib, an HSP90 inhibitor, has been shown to induce necroptosis and destabilize HIF-1α under hypoxic conditions, enhancing the therapeutic efficacy of sorafenib. Consequently, the co-administration of ganetespib and sorafenib may trigger necroptosis, destabilize HIF-1α, inhibit macrophages, and produce anti-angiogenic effects [31]. IgM monoclonal antibodies, particularly clone M6-1D4, target CD147 and cause significant swelling in HCC cells. Inhibition of MLKL decreases HepG2 cell susceptibility to necroptosis when treated with M6-1D4, suggesting an innovative approach to cancer treatment that triggers necroptosis [32].

Moreover, ZZW-115, a NUPR1 inhibitor, induces apoptosis and necroptosis in HCC cells, resulting in pronounced mitochondrial dysfunction and a marked reduction in ATP levels, making it a viable pharmacological candidate for patients with HCC [33]. In addition, shikonin, a small molecule from the Chinese herb Lithospermum, induces necroptosis in tumor cells when apoptosis is inhibited; although sonodynamic therapy (SDT) targets tumors through ultrasound cavitation and the chemical effects of singlet oxygen, hydroxyl radicals, and sonosensitizers like indocyanine green (ICG) to produce reactive oxygen species (ROS), SDT alone has limited impact on apoptosis-resistant tumors. However, combining ICG-nanobubble-mediated SDT with shikonin enhances necroptosis in HCC by increasing ROS, which regulates the RIPK1/RIPK3 necroptotic pathway [34].

Although necroptosis contributes to inflammation, fibrosis, and carcinogenesis in the liver, its induction in existing HCC cells is lethal. Harnessing the necroptotic pathway, either alone or in conjunction with other anticancer therapeutics, has broad potential for HCC treatment (Fig. 2).

### Hepatocellular carcinoma and pyroptosis

#### Molecular pathways of pyroptosis

Pyroptosis was originally identified in Shigella flexneri-infected macrophages, which is characterized by inflammasome activation and the subsequent release of pro-inflammatory cytokines such as interleukin-1β (IL-1β) and interleukin-18 (IL-18) [35]. The inflammasomes responsible for pyroptosis are categorized into classical caspase-1-dependent and non-classical caspase-11-dependent types. Activation of these inflammasomes results in the cleavage of gasdermin D (GSDMD) into a 22kDa C-terminal fragment (GSDMD-C) and a 31kDa N-terminal fragment (GSDMD-N) [36]. GSDMD-N swiftly moves to the inner layer of the plasma membrane where it binds to phospholipids and initiates pore formation, ultimately causing membrane rupture. Conversely, GSDMD-C inhibits the pore-forming ability of GSDMD-N [37]. Although caspase-3 is known for its role in apoptosis, it has also been implicated in pyroptosis through the cleavage of GSDME, generating pores in the cell membrane [38].

**Fig. 3:**
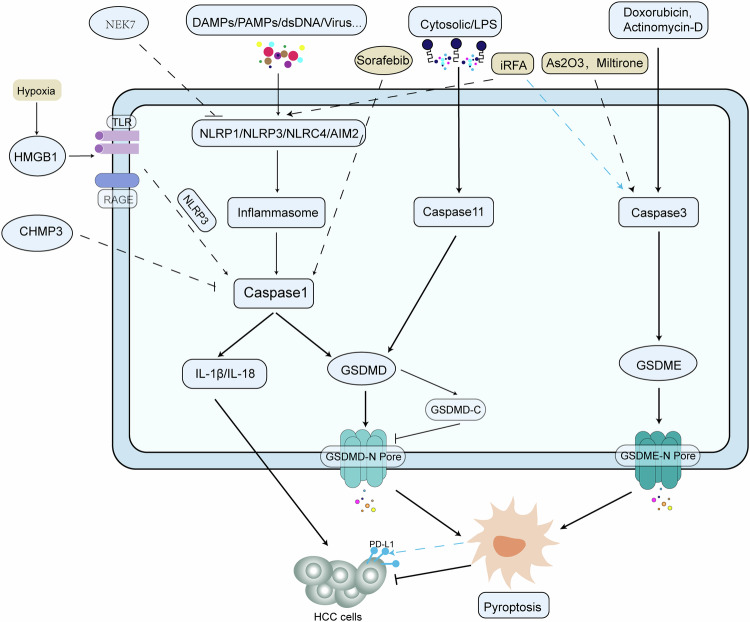
The pyroptosis mechanism in HCC. Pyroptosis is triggered by inflammasome activation, resulting in the cleavage of GSDMD by CASP1 or CASP11 to form GSDMD-C and GSDMD-N. GSDMD-N creates pores in the cell membrane, causing it to rupture, while GSDMD-C inhibits this process. Caspase-3 can also induce pyroptosis by cleaving GSDME to generate GSDME-N pores on the cell membrane. Pyroptosis helps prevent tumor growth and is controlled by various molecules and drugs. However, the Caspase-1 pathway of pyroptosis can increase levels of IL-1β and IL-18, promoting HCC progression. Additionally, iRFA-induced pyroptosis can lead to the production of PD-L1, aiding in the immune evasion of liver cancer cells.

Pyroptosis exerts a dual effect on HCC. On one hand, hypoxic HCC cells can activate caspase-1 via the TLR4 and receptor of advanced glycation endproducts (RAGE) signaling pathways due to high-mobility group box 1 (HMGB1) induction, promoting invasion and metastasis through the upregulation of pro-inflammatory factors including IL-1β and IL-18 [39]. In contrast, pyroptosis predominantly inhibits HCC progression. For instance, charged multivesicular body protein 3 (CHMP3), a part of the endosomal sorting complex required for transport (ESCRT), is overexpressed in HCC. Its knockdown may trigger the caspase-1-mediated pyroptosis pathway, thus impeding HCC progression, an effect reversible by caspase-1 inhibitors such as AYC [40]. Furthermore, silencing of never-in-mitosis gene a-related kinase 7 (NEK7), a serine/threonine kinase, in HCC cells markedly increases pyroptosis markers such as NOD-like receptor thermal protein domain-associated protein 3 (NLRP3), caspase-1, and GSDMD, encouraging pyroptosis and reducing tumor-stroma interactions [41] (Fig. 3).

#### The role of pyroptosis in anti-tumor therapy

Anti-tumor agents against HCC elicit antineoplastic responses by inducing pyroptosis. For instance, sorafenib has been reported to induce pyroptosis in macrophages by targeting caspase-1-dependent inflammasomes, thereby activating cytotoxic natural killer (NK) cells and precipitating tumor cell death [42]. Arsenic trioxide (As2O3) is known to initiate pyroptosis in HCC by activating caspase-3-mediated cleavage of GSDME, subsequently halting tumor progression [43]. Similarly, miltirone, a quinone derivative extracted from S. miltiorrhiza, induces pyroptosis in HCC cells by promoting GSDME hydrolysis and caspase-3 activation, thus facilitating tumor cell death [44]. However, Liang et al. found that incomplete radiofrequency ablation promotes cell pyroptosis via the caspase-3/GSDME pathway, leading to increased programmed cell death 1 ligand-1 (PD-L1) expression in residual HCC cells and consequent resistance to anti-PD-L1 immunotherapy by bolstering T cell exhaustion [45].

Hence, pyroptosis and its associated molecules exhibit both pro- and anti-tumorigenic effects. Moreover, anti-tumor drugs may stimulate pyroptosis to combat tumor cells, and incomplete ablation can foster HCC resistance to anti-PD-L1 through pyroptosis (Fig. 3).

### Hepatocellular carcinoma and ferroptosis

#### The involvement of ferroptosis in various regulatory mechanisms

Ferroptosis is intricately associated with lipid peroxidation, resulting from iron overload and oxidative stress. Morphologically, ferroptosis is characterized by the shrinkage of mitochondrial volume, increased mitochondrial membrane density, disappearance or reduction of mitochondrial cristae, rupture of the mitochondrial outer membrane, and maintenance of an intact cellular membrane with a normal-sized cell nucleus devoid of chromatin condensation. Biochemically, this process is marked by glutathione (GSH) depletion, lower GSH peroxidase 4 (GPX4) activity, reduced cellular antioxidant capacity, enhanced lipid peroxidation, metabolic dysfunction, and increased levels of lipid ROS, which collectively promote ferroptosis [46, 47]. The regulation of ferroptosis encompasses three primary suppressing systems: the cysteine/GSH/GPX4 axis (XC-system), the NAD(P)H/FSP1/CoQ10 system, and the GCH1/BH4/DHFR system [48].

In HCC, ferroptosis is influenced by a multitude of factors, including post-translational modifications, such as phosphorylation, acetylation, and methylation, along with non-coding RNAs, mitochondrial proteins, and endoplasmic reticulum (ER) stress. These factors predominantly affect regulatory systems. For instance, the activation of the insulin-like growth factor 1 receptor (IGF1R) in HCC cells increases GPX4 levels, and creatine kinase B (CKB) phosphorylates GPX4 to inhibit its degradation, thereby inhibiting ferroptosis and facilitating tumorigenesis [49]. Similarly, hydroxymethylglutaryl-CoA lyase (HMGCL) influences histone H3 lysine 9 (H3K9) acetylation via β-hydroxybutyrate, which regulates dipeptidyl peptidase 4 (DPP4) expression in a dose-dependent manner, promoting ferroptosis in HCC cells [50]. The Wilm’s tumor 1-associated protein (WTAP), a known m6A RNA methyltransferase, fosters autophagic ferroptosis through YTHDC2-associated mechanisms [51]. Additionally, high-density lipoprotein binding protein (HDLBP) in HCC reduces ferroptosis susceptibility by binding to cytoplasmic ferroptosis-related long non-coding RNA (lncRNA) lncFAL, precluding Tripartite Motif-Containing Protein 69 (TRIM69)-dependent degradation of ferroptosis suppressor protein 1 (FSP1) [52]. Mir-612 modulates coenzyme Q10 (CoQ10) expression through the HADHA-mediated mevalonate pathway, elevating cellular polyunsaturated fatty acid levels (PUFA) and lipid peroxidation, thereby promoting ferroptosis in HCC cells [53]. The mitochondrial translocator protein (TSPO) upregulates antioxidant genes and PD-L1 expression via the p62/KEAP1/Nrf2 pathway, offering resistance to ferroptosis and suppressing anti-tumor immunity in HCC [54].

**Fig. 4:**
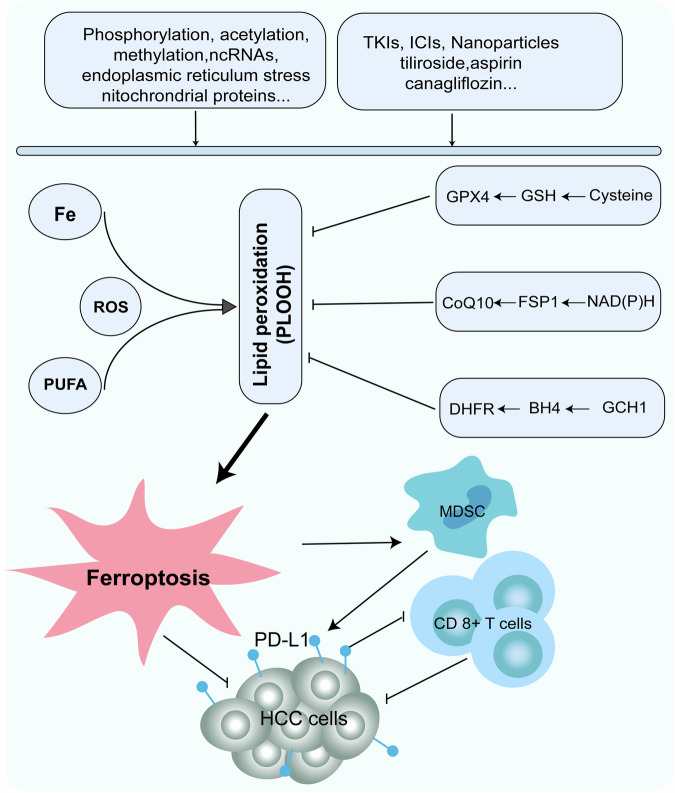
The ferroptosis mechanism in HCC. Ferroptosis is primarily induced by lipid peroxidation driven by iron overload and oxidative stress and is regulated by three major systems: the cysteine/GSH/GPX4 axis, the NAD(P)H/FSP1/CoQ10 system, and the GCH1/BH4/DHFR system. Ferroptosis is also regulated by various factors, including phosphorylation, acetylation, methylation, ncRNAs, ER stress, and mitochondrial proteins, thereby modulating the progression of HCC. Anti-tumor treatments for HCC operate by modulating these pathways, and disruption of these pathways may lead to drug resistance.

All the ferroptosis-related processes mentioned above are involved in anti-tumor activity. However, activating ferroptosis signaling may also induce adaptive tolerance of tumor cells to endoplasmic reticulum stress (ERS), allowing them to evade ERS-induced cell death. Mechanistically, it has been found that activating ferroptosis signaling promotes the formation and secretion of exosomes containing misfolded and unfolded proteins, alleviating ERS and supporting tumor cell survival [55] (Fig. 4).

#### Ferroptosis and anti-tumor treatment

Multi-target tyrosine kinase inhibitors (TKIs) have demonstrated anti-tumor effects through the induction of ferroptosis; however, the development of resistance in HCC to TKIs can occur upon inhibition of the ferroptosis pathway [56]. Sorafenib, a ferroptosis inducer, disrupts cystine uptake by impeding the XC-system, diminishing GSH biosynthesis, and amplifying mitochondrial ROS production, all of which contribute to ferroptosis in HCC [57]. Nonetheless, mitochondrial dysfunction precipitated by sorafenib may activate the PI3K/RAC1/PAK1 signaling pathway, leading to an influx of liver cancer-associated macrophages that provide intracellular cysteine, thereby conferring sorafenib resistance via ferroptosis inhibition [58]. Additionally, the hepatitis B virus (HBV) can suppress ferroptosis by decreasing intracellular iron (Fe2 + ) concentrations and upregulating GPX4 through the SRSF2/PCLAF pathway, which diminishes HCC sensitivity to sorafenib [59]. Furthermore, HBXIP and SLC27A4 provoke an accumulation of free fatty acids in HCC cells via distinct pathways, thereby hindering sorafenib-evoked ferroptosis and promoting drug resistance [60, 61]. Various other factors, including the transcription factors YAP/TAZ, lncRNA DUXAP8, fatty acid synthase (FASN), and ABCC5, may also inhibit sorafenib-induced ferroptosis by elevating SLC7A11 expression and reducing HCC sensitivity to ferroptosis [62–65]. Consequently, combined antiviral therapy for HBV targeting these aberrant ferroptosis-related factors or combining ferroptosis inducers can enhance the drug sensitivity of agents, such as sorafenib, by promoting ferroptosis, thereby inhibiting HCC progression.

Research has shown that nanomaterials have great potential for the combined delivery of ferroptosis inducers and anti-tumor drugs for HCC. For instance, Xiao et al. employed cell membrane-derived nanovesicles charged with transferrin-protein-Fe3+ (TF NVs) and sorafenib (SOR@TF-Fe3+ NVs) to potentiate ferroptosis and circumvent sorafenib resistance [66]. Moreover, the synthesis of pH-responsive amphiphilic hyperbranched polyglycerol (HDP) through co-grafting has proven effective in co-delivering sorafenib and siRNA, thereby augmenting the drug’s anti-tumor efficacy [67]. In addition, other TKIs have also been found to have anti-tumor effects through ferroptosis pathways. For example, the combination of donafenib and GSK-J4, an inhibitor of histone demethylase KDM6A/B, synergistically enhances HMOX1 expression, increases intracellular Fe2+ levels, and induces ferroptosis in HCC cells, thereby exerting anti-tumor activity [68].

The modulation of ferroptosis has been demonstrated to improve the immunosuppressive TME and enhance the response of HCC cells to immunotherapy. Immune therapy-activated cytotoxic T cells release interferon-gamma (IFN-γ), antagonize the Xc-system, and drive mitochondrial lipid peroxidation, leading to the ferroptosis of HCC cells [69]. Ferroptosis leads to the release of tumor-associated antigens (TAA), enhancing TME immunogenicity and the overall efficacy of immunotherapy [70]. Nevertheless, ferroptosis induces the activation of CD8 + T cells, which can be mitigated by increased PD-L1 expression in tumor cells and HMGB1-mediated infiltration of myeloid-derived suppressor cells (MDSC). Co-therapy of ferroptosis inducers and MDSC inhibitors can sensitize HCC and its metastases to immune checkpoint inhibitors (ICIs) therapy [71]. Additionally, targeting phosphoglycerate mutase 1 (PGAM1) has shown promise in promoting ferroptosis and enhancing CD8 + T cell infiltration, which, when combined with ICIs, could amplify anti-tumor effects [72]. Moreover, the suppression of apolipoprotein C1, a crucial lipid metabolism protein, induces M1 polarization through the ferroptosis pathway, thereby reconfiguring the immunosuppressive TME and improving the outcomes of ICIs in HCC [73]. Collectively, these findings indicate that combining ferroptosis with immunotherapy may confer synergistic benefits in the treatment of HCC.

In addition to targeted immunotherapy, various other anti-tumor agents can induce ferroptosis to exert their therapeutic effects. For instance, canagliflozin triggers ferroptosis by simultaneously disrupting glycolysis and glutamine metabolism, thus enhancing the sensitivity of HCC cells to cisplatin treatment [74]. Aspirin also has the potential to impede HCC progression by downregulating the transcription of SLC7A11, a crucial ferroptosis regulator activated by NF-κB signaling [75]. Tiliroside, a robust tank-binding kinase-1 (TBK1) inhibitor, has emerged as a promising natural compound capable of augmenting the effectiveness of sorafenib in HCC therapy by targeting TBK1 and inducing ferroptosis [76].

Consequently, ferroptotic pathways and associated molecules are important for the etiology and progression of HCC. They are influenced by a variety of cytokines and genetic alterations and are intertwined with numerous pathways and resistance mechanisms of multiple anti-tumor drugs in HCC (Fig. 4).

### Hepatocellular carcinoma and immunogenic cell death

#### Molecular mechanism of immunogenic cell death

**Fig. 5:**
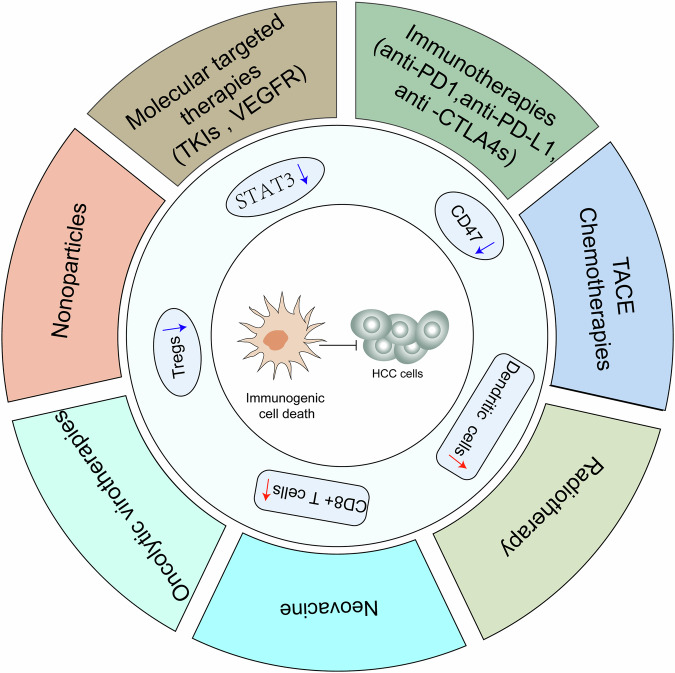
The ICD mechanism in HCC. ICD refers to the initiation of innate and adaptive immune responses triggered by selected anticancer therapies, resulting in the production of specific CD8 + T lymphocytes that induce cell death through immune cytotoxicity, and concurrently activating DCs to enhance anti-tumor immunity. Inhibition of STAT3 and CD47 aslso play an important role in inducing the ICD of HCC cells.

ICD refers to the activation of the innate and adaptive immune responses triggered by selected anticancer therapies. This process is mediated by the release of damage-associated molecular patterns (DAMPs) which are subsequently recognized by pattern recognition receptors (PRRs), leading to the generation of specific CD8 + T lymphocytes that induce cell death via immune cytotoxic effects [77, 78]. The signal transducer and activator of transcription 3 (STAT3) is pivotal for inducing ICD in HCC. The inhibition of STAT3 could induce ICD of HCC cells via translocation of the “eat me” molecule calreticulin to the cell surface and a significant reduction in the expression of the “don’t eat me” molecule leukocyte surface antigen CD47. Furthermore, targeting STAT3 bolsters dendritic cell (DCs) activation, augments macrophage recognition and phagocytosis in HCC cells, and cultivates anti-HCC immune memory, along with the accumulation of key anti-tumor effector CD8 + T cells within the TME [79] (Fig. 5).

#### Immunogenic cell death in anti-tumor therapies

Various interventions, including targeted therapy, chemotherapy, and ablation, can elicit ICD in HCC cells, resulting in anti-tumor effects. For instance, lenvatinib facilitates ICD in HCC by elevating PD-L1 expression via TLR4 and stimulating cell death via TLR3 [80]. Oxaliplatin bolsters ICD-related biomarkers, augments CD8 + T cell and mature DCs populations, and diminishes Treg cells [81]. Transarterial chemoembolization (TACE) is correlated with reduced tumor-infiltrating Tregs, attenuated immune exhaustion, cell cytotoxicity, and marked pro-inflammatory pathway activation [82]. Cryoablation triggers ICD, activates DCs, and strengthens anti-tumor immunity in HCC, potentially converting “cold” tumors into “hot” tumors [83]. These anti-tumor therapeutic strategies, combined with ICIs, can enhance immune cytotoxic effects on liver cancer cells, resulting in a synergistic effect.

Moreover, emerging treatments, such as nanomaterials and tumor neoantigen vaccines, exhibit considerable promise in preclinical trials for inducing ICDs in HCC, with notable anti-tumor effects. Zhao et al. developed a compact nanofiber patch comprising germanium phosphide (GeP) and anlotinib, serving as a multifaceted platform for an integrated trimodal treatment strategy that combines molecularly targeted photothermal therapy with ICIs. The GeP-mediated increase in temperature under near-infrared light potentiates controlled anlotinib release, reduces vascular endothelial growth factor receptor expression, induces ICD in neoplastic cells, and promotes DCs maturation. The simultaneous application of ICI therapy enhances therapeutic efficacy, ultimately impeding the progression and metastasis of HCC [84]. In addition, a neoantigen vaccine (NeoVAC) coupled with anti-PD-1 induced robust anti-tumor responses and persistent tumor-specific immune memory in HCC by enhancing CD8+ Trm infiltration, making it a potential immunotherapy target in HCC [85]. Therefore, ICD plays an integral role in HCC anti-tumor therapy (Fig. 5).

### Hepatocellular carcinoma and other programmed cell deaths

Other forms of PCD, such as NETosis, parthanatosis, entosis, and LCD, may also influence the occurrence and progression of HCC. However, related studies are scarce and lack an in-depth investigation of these mechanisms. NETosis facilitates HCC metastasis through neutrophil extracellular traps (NETs) [86]. DNASE1L3 deficiency aggravates NET-DNA-driven HCC invasion in diabetic patients via the cGAS and non-canonical NF-kB pathways [87]. Parthanatos, a PARP1-dependent pathway, is triggered by oxidative DNA damage, leading to AIFM1’s translocation from mitochondria to the nucleus and consequently causing chromatin disassembly [88]. MLKL deficiency hampers Mg2+ exchange between the ER and mitochondria, inciting ER dysfunction and oxidative stress, thereby increasing the vulnerability of HCC cells to parthanatos [89]. Entosis, which is directed by Rnd3 deficiency and the RhoA/ROCK pathway, contributes to HCC progression by promoting homotypic cell engulfment and death via cathepsin B-mediated pathways [90, 91].

Moreover, LCD, which is characterized by lysosomal rupture, is triggered by lysosomal hydrolase release [92]. Polyphyllin D, a lysosome-targeting agent, disrupts this pathway and has anti-HCC properties [93]. Autosis is characterized by cellular adhesion augmentation, nuclear contour alterations, and ER disintegration. HDAC6 downregulation in HCC facilitates this process through the JNK/Beclin 1 pathway [94, 95]. Oxeiptosis, an oxidative stress-induced pathway independent of caspases, is activated by the KEAP1-PGAM5-AIFM1 axis [96]. The combination of phenethyl isothiocyanate and dasatinib synergistically reduced HCC growth through cell cycle arrest and oxeiptosis [97]. Cuproptosis, caused by Cu-induced mitochondrial perturbations, is sensitized by ARID1A deficiency via a metabolic shift that affects HCC cell survival [98, 99]. Disulfidiptosis, characterized by an overabundance of cysteine and atypical disulfide bonds within cytoskeletal proteins resulting from increased SLC7A11 expression in low-glucose conditions, has the potential to serve as a significant biomarker for immune infiltration, drug response, and prognosis in HCC [100, 101].

## Discussion and perspective

Diverse PCD pathways display unique characteristics based on their underlying molecular mechanisms. In HCC, these pathways are not isolated but coexist and interact synergistically. The aforementioned information illustrates that an anti-tumor drug can affect multiple PCD signaling pathways simultaneously, and several drugs can regulate a single PCD signaling pathway concurrently (Table 1). However, the PCD pathway and its regulatory components, which play critical roles in HCC, have not yet been elucidated. The dominant PCD pathway may vary under different environmental conditions. For instance, Wataru Jomen et al. found that sorafenib monotherapy for HCC predominantly induced necroptosis at all concentrations, with high concentrations also inducing ferroptosis, while deferasirox monotherapy at high concentrations favored apoptosis over necroptosis. In combination therapy, ferroptosis was suppressed, apoptosis and necroptosis were dominant, and NF-κB expression in the nucleus was suppressed across all treatments, with the combination therapy showing additive anti-tumor effects through programmed cell deaths and NF-κB signal modification [102]. Moreover, the interaction between ferroptosis and pyroptosis in HCC highlights the key role of 3-hydroxy-3-methylglutaryl-coenzyme A reductase (HMGCR), which transitions between mitochondria during ferroptosis and the ER when pyroptosis is induced. HMGCR deubiquitination is regulated by BRCC36 in a manner dependent on its deubiquitinase activity, inhibiting ferroptosis and enhancing pyroptosis [103].

**Table 1:** The therapeutic potential of drugs that regulate various PCDs in HCC.

Drugs/inhibitors	Cell death	Mechanism	Ref
Sorafenib	Apoptosis ↑	BAX/BAK stimulation via BH3-only protein.	[20]
	Necroptosis ↓	Hypoxia, HSP90 increase, RIPK1/RIPK3/MLKL complex inhibition, autophagy degradation.	[31]
	Pyroptosis ↑	Caspase-1-dependent pyroptosis of macrophages, NK cell activation.	[42]
	Ferroptosis↑	XC-system inhibition, mitochondrial ROS production.	[57]
	Ferroptosis↓	Mitochondrial dysfunction, PI3K/RAC1/PAK1 pathway activation, influx of TAM that provides cysteine.	[58]
	Autosis ↑	SHP1-STAT3-Mcl1-Beclin 1 pathway activation.	[104]
Donafenib/ GSK-J4	Ferroptosis↑	HMOX1 expression, intracellular Fe2+ level elevation.	[68]
Sunitinib/ cisplatin	Apoptosis↑	ERK stimulation, Bcl-2 downregulation, Bax upregulation, caspase-9/8 levels elevation.	[105]
	Necroptosis ↓	Inhibition of pRIPK3/RIPK3.	[105]
Lenvatinib	ICD ↑	PD-L1 elevation via TLR4, cell death through TLR3.	[80]
PEITC/dasatinib	Oxeiptosis ↑	ROS production, mitotic catastrophe, cell cycle arrest.	[97]
Oxaliplatin	ICD ↑	T cell accumulation, DCs maturation, synergizing with anti-PD-1.	[81]
TACE	ICD ↑	Tregs reduction, immune exhaustion cell cytotoxicity attenuation, pro-inflammatory activation.	[82]
Cryo-thermal ablation	ICD ↑	DCs activattion, anti-tumor immunity strengthening, “cold” tumors into “hot” tumors conversion.	[83]
Incomplete radiofrequency ablation	Pyroptosis ↓	HSP70 upregulation, NLRP3 inflammasome inhibition.	[106]
	Pyroptosis ↑	Caspase-3-mediated cleavage of GSDME.	[45]
M6-1D4	Necroptosis ↑	MLKL Phosphorylation independent of RIPK1/3 mechanism.	[32]
ZZW-115	Apoptosis ↑	Cell death can be inhibited by Z-VAD-FMK.	[33]
	Necroptosis↑	Cell death can be inhibited by Necrostatin-1.	[33]
IFN-γ	Ferroptosis ↑	Glutathione depletion and lipid peroxidation increase.	[69]
CANA	Ferroptosis ↑	PKM2-c-Myc complex-mediated glutamine starvation.	[74]
Aspirin	Ferroptosis ↑	Restricting NF-κB p65-activated SLC7A11 transcription.	[75]
Miltirone	Pyroptosis ↑	ROS-mediated damage, MEK/ERK1/2 inhibition, GSDME promotion.	[44]
Tiliroside	Ferroptosis↑	TBK1 inhibition, Nrf2 ubiquitination and degradation, the oxidative stress balance disruption.	[76]
Polyphyllin D	LCD ↑	Puncturing hypertrophic lysosomes by targeting acid sphingomyelinase.	[93]
ICG-NB-SDT/ shikonin	Necroptosis↑	ROS generation, RIPK1/RIPK3 signaling.	[34]
AsO3-NPs	Pyroptosis ↑	Caspase-3-mediated cleavage of GSDME, GSDME-N upregulation.	[43]
SOR@TF-Fe3+NVs	Ferroptosis ↑	Iron transport metabolism promotion by TFRC/TF-Fe3+, SOR efficacy enhancement by SLC7A11 inhibition.	[66]
Gep-AL-ICI nanofiber patch	ICD ↑	Vascular-related receptors diminishment, fostering ICI in tumor cells, and maturing DCs.	[84]
NeoVAC	ICD ↑	Enhancing CD8+ Trm infiltration.	[85]

This review summarizes various PCD modalities pertinent to HCC and elucidates their significance in disease onset and progression. Furthermore, we explore the utilization of PCD and related biomarkers for the treatment of HCC. Apoptosis, necroptosis, pyroptosis, ferroptosis, and ICD are the five most extensively studied pathways of PCD in HCC. These cell death mechanisms are closely associated with HCC development, and various anti-tumor agents target these pathways. The absence of PCD pathways may contribute to drug resistance in HCC, and key molecules in PCD pathways may serve as potential therapeutic targets for HCC. Enhancing HCC’s drug responsiveness may be achieved by combining PCD inducers with anticancer agents, while the co-delivery of PCD inducers and anti-tumor drugs using nanoparticles promises to substantially improve treatment efficacy. However, a deeper understanding of the molecular mechanisms and principal regulatory factors governing these cell death variations is imperative for advancements in HCC therapy.
